# Interneuron Functional Diversity in the Mouse Accessory Olfactory Bulb

**DOI:** 10.1523/ENEURO.0058-19.2019

**Published:** 2019-08-13

**Authors:** Marina A. Maksimova, Hillary L. Cansler, Kelsey E. Zuk, Jennifer M. Torres, Dylan J. Roberts, Julian P. Meeks

**Affiliations:** 1 Department of Neuroscience, University of Texas Southwestern Medical Center, Dallas, TX 75390; 2 Department of Neurology and Neurotherapeutics, University of Texas Southwestern Medical Center, Dallas, TX 75390

**Keywords:** accessory olfactory bulb, cell types, excitability, interneuron

## Abstract

In the mouse accessory olfactory bulb (AOB), inhibitory interneurons play an essential role in gating behaviors elicited by sensory exposure to social odors. Several morphological classes have been described, but the full complement of interneurons remains incomplete. In order to develop a more comprehensive view of interneuron function in the AOB, we performed targeted patch clamp recordings from partially overlapping subsets of genetically labeled and morphologically defined interneuron types. *Gad2* (GAD65), *Calb2* (calretinin), and *Cort* (cortistatin)-cre mouse lines were used to drive selective expression of tdTomato in AOB interneurons. *Gad2* and *Calb2-*labeled interneurons were found in the internal, external, and glomerular (GL) layers, whereas *Cort*-labeled interneurons were enriched within the lateral olfactory tract (LOT) and external cellular layer (ECL). We found that external granule cells (EGCs) from all genetically labeled subpopulations possessed intrinsic functional differences that allowed them to be readily distinguished from internal granule cells (IGCs). EGCs showed stronger voltage-gated Na^+^ and non-inactivating voltage-gated K^+^ currents, decreased I_H_ currents, and robust excitatory synaptic input. These specific intrinsic properties did not correspond to any genetically labeled type, suggesting that transcriptional heterogeneity among EGCs and IGCs is not correlated with expression of these particular marker genes. Intrinsic heterogeneity was also seen among AOB juxtaglomerular cells (JGCs), with a major subset of *Calb2*-labeled JGCs exhibiting spontaneous and depolarization-evoked plateau potentials. These data identify specific physiological features of AOB interneurons types that will assist in future studies of AOB function.

## Significance Statement

The mouse accessory olfactory bulb (AOB) plays a critical role in processing social chemosensory information. Several morphologically identified types of AOB inhibitory interneurons are thought to refine and restrict information flow from the AOB to its downstream targets in the limbic system. However, little is known about the electrophysiological and transcriptional diversity among AOB interneuron types. We systematically investigated intrinsic electrophysiological diversity across 5 AOB cell populations in three transgenic mouse lines. Analysis of 26 intrinsic physiologic features revealed feature combinations associated with identified morphologic AOB cell types, but few associated with the transgenic lines we studied. The results provide quantitative information about functional diversity in AOB interneurons and provide an improved foundation for future studies of AOB circuit function.

## Introduction

The mammalian brain utilizes a diverse complement of interneuron types to achieve its overall function. In well-studied neural circuits like the hippocampus, neocortex, and retina, interneuron types have been painstakingly identified and catalogued over a period of decades using a combination of morphologic and electrophysiological metrics (Freund and Buzsáki, 1996; Somogyi and Klausberger, 2005; Burton, 2017). Transgenic and viral technologies allow selective labeling and manipulation of specific interneuron types in olfactory circuits, facilitating research into functional subdivisions among genetically defined types (Parrish-Aungst et al., 2007; Eyre et al., 2009; Huang et al., 2013; Kato et al., 2013; Miyamichi et al., 2013; Tatti et al., 2014; Burton et al., 2017). Importantly, many neural circuits, including the accessory olfactory bulb (AOB), lack a comprehensive “taxome” that links genetic, morphologic, and functional properties of interneurons. A more complete and quantitative description of the interneuron classes in the AOB would provide the ground knowledge useful for building a deeper understanding of AOB circuit function.

The mouse AOB, the first neural circuit in the mammalian accessory olfactory system (AOS; also called the vomeronasal system), is a critical regulator of mouse behavior. Although analogous cell types appear to be present in the main olfactory bulb (MOB), it is clear that the AOB and MOB have distinct organizations (Jia et al., 1999; Taniguchi and Kaba, 2001; Araneda and Firestein, 2006; Castro et al., 2007; Shpak et al., 2012; Smith et al., 2015; Gorin et al., 2016). In the quest to achieve a better understanding of AOB function and its impact on social behavior, establishing a taxome of AOB interneuron is an important step.

A comprehensive morphologic survey of the AOB neuronal types identified up to 6 distinct interneuron types in the AOB (Larriva-Sahd, 2008). Importantly, among the identified cell types in the AOB is a large set of spiny interneurons called external granule cells (EGCs) that have not been studied in depth. EGCs reside in the external cellular layer (ECL) alongside mitral cells (MCs), the only projection neurons of the AOB. EGCs, like juxtaglomerular cells (JGCs) and IGCs, appear to lack axons, and are thought to form reciprocal dendro-dendritic synapses with MCs. EGCs have elaborate radial dendritic arbors studded with long-necked gemmules or spines, and as such likely make extensive lateral connections in the circuit. However, physiologic data concerning the intrinsic and synaptic features of these cells are needed to build informed hypotheses about their function.

Here, we present electrophysiological data recorded from 150 genetically and morphologically defined AOB interneurons across all of the major AOB sublaminae, including JGCs, EGCs, and IGCs. To avoid explicit bias in our choice of interneurons from which to record, and to test for potential links between marker gene expression and function, we made targeted recordings from largely non-overlapping sets of genetically labeled AOB interneurons (via *Calb2-cre*, *Gad2-cre*, and *Cort-cre* transgenic mice). We used a battery of intrinsic electrophysiological assays and multidimensional analyses to identify functionally similar groups. We found that IGCs, EGCs, and JGCs possess characteristic physiologic features that are largely invariant across genetically labeled groups. EGCs, in particular, possess a combination of physiologic features that indicate a potential role in broad MC inhibition. These data show clear functional subdivisions across morphologically defined AOB interneuron classes, but also reveal that physiologic features of these interneurons are shared across genetically distinct types.

## Materials and Methods

### Mice

A total of 75 adult male and female mice aged 6–12 weeks were used in this study. All animals were housed in the care of the University of Texas Southwestern Medical Center Animal Resource Center, and were given food and water *ad libitum* before euthanasia. All studies were performed in accordance with the University of Texas Southwestern Medical Center Institutional Animal Care and Use Committee. Animals were housed with a 12/12 h light/dark cycle, and slices were taken at Zeitgeber time of 20–24 h, corresponding to the latter part of time in darkness for the mice.

To generate offspring expressing the fluorescent protein tdTomato in genetically restricted types, we crossed *Gad2^tm2(cre)Zjh^/J* (Jackson Labs stock 010802, “*Gad2-cre*”), *B6(Cg)-Calb2^tm1(cre)Zjh^/J* (Jackson Labs stock 010774, “*Calb2-cre*”), or *Cort^tm1(cre)Zjh^/J* (Jackson Labs stock 010910, “*Cort-cre*”) animals to *B6;129S6-Gt(ROSA)26Sor^tm9(CAG-tdTomato)Hze^/J* (Jackson Labs Stock 007905, *ROSA26-loxP-STOP-loxP-tdTomato* or “Ai9”) animals (Madisen et al., 2010; Taniguchi et al., 2011). Genotyping was performed by Transnetyx, Inc. All transgenic animals were heterozygous for both knock-in transgenes. All animals were backcrossed for multiple generations with C57Bl/6J animals before arrival at our facility.

### Immunohistochemistry

Animals were anesthetized lightly with isofluorane, then injected with ketamine/xylazine cocktail (120 mg/kg ketamine, 16 mg/kg xylazine) to induce deep anesthesia. Animals were transcardially perfused with cold PBS followed by 4% paraformaldehyde in PBS (PFA-PBS). Brains were then removed from the skull, bisected at the midline, and post-fixed in PFA-PBS for 2–12 h. Hemispheres were then cryoprotected in PBS with 25% w/v sucrose for 12–24 h. Hemispheres were aligned with the sagittal plane, then frozen in a block of OCT compound (TissueTek) for 1–2 h, then sectioned on a cryostat (Leica CM3050 S) at 20 µm.

Sections were rinsed 3x with PBS in 24-well plates, then permeabilized by incubating for 2 h in PBS containing 0.3% Triton X-100. Sections were blocked in PBS containing 0.1% Triton X-100 containing 10% goat serum (“primary block”) for 2–4 h. Primary antibodies were diluted into primary block, and sections were incubated overnight at 4°C, followed by a 4× rinse in PBS. Secondary antibodies were diluted into PBS containing 0.1% Triton X-100 and 5% goat serum (“secondary block”). Sections were exposed to secondary antibodies for 2 h, followed by a final 4× PBS rinse. Negative controls omitted primary antibodies (data not shown). Sections were mounted on slides, coverslipped using Fluoromount-G (SouthernBiotech) and sealed with nail polish. Immunostaining for calretinin (Abcam ab702) largely but incompletely overlapped with *Calb2*-tdTomato expression, indicating that our strategy of Cre-driver x Cre-dependent reporter may have labeled cells that transiently expressed *Calb2* during development (data not shown). Commercially available selective antibodies for GAD65 (Abcam ab26113) and cortistatin (Santa Cruz Biotechnology SC-68890) were evaluated but deemed unsuitable for cellular quantification.

Images of immunostained sections were taken at 40× (1.3 NA) on a Zeiss LSM 510 confocal microscope. All images for a given staining batch were imaged with matched laser power and photomultiplier tube gain. For display purposes, maximum projection images of 5–10 frames (1 µm per frame) were created with ImageJ, then stitched manually in Adobe Photoshop. Quantification of cell density was performed using ImageJ.

### Acute slice preparation

Standard artificial CSF (aCSF) contained the following: 125 mM NaCl, 2.5 mM KCl, 2 mM CaCl_2_, 1 mM MgCl_2_, 25 mM NaHCO_3_, 1.25 mM NaH_2_PO_4_, 3 mM myo-inositol, 2 mM Na-pyruvate, 0.4 mM Na-ascorbate, and 25 mM glucose. Animals were anesthetized lightly with isofluorane and then rapidly decapitated into ice-cold aCSF with an additional 9 mM MgCl_2_ (10 mM MgCl_2_ total). Brains were extracted in cold slicing buffer, and the anterior portion of the brain containing the olfactory bulbs and frontal neocortex was isolated from the rest of the brain. This anterior brain tissue was separated into two hemispheres and then embedded in aCSF containing 4% agarose in aCSF. The tissue was then affixed to an angled slicing tray with tissue glue in a vibrating microtome (Leica). Slices were cut at an angle of at ∼12° off-sagittal such that the caudal/medial aspect of the olfactory bulbs was cut first. We find this angle to better preserve the long IGC neurites. After vibrosectioning, slices were immediately placed into a holding chamber containing standard aCSF containing 0.5 mM kynurenic acid. Slices were allowed to recover for at least 30 min before placing in the recording chamber.

### Electrophysiology

Electrophysiological experiments were performed using a custom epifluorescence upright microscope (Nikon). Brain slices were placed in a large volume tissue chamber (Warner Instruments) warmed to 33°C by a temperature controller (Warner Instruments). tdTomato-expressing neurons were identified by imaging with a 40× objective (Olympus) and digital camera (Point Gray) with dim fluorescent illumination (X-Cite 200DC). Patch electrodes were pulled on a horizontal Flaming/Brown-style puller (P1000, Sutter Instruments) using thin-walled borosilicate glass (TW150, World Precision Instruments). Pipette resistances ranged from 6 to 10 MΩ, and optimal recordings were achieved with 8- to 9-MΩ electrodes. Internal solution for intrinsic characterization experiments contained the following: 115 mM K-gluconate, 20 mM KCl, 10 mM HEPES, 2 mM EGTA, 2 mM MgATP, 0.3 mM Na_2_GTP, and 10 mM Na-phosphocreatine, with a pH of 7.37. Internal solution for voltage clamp experiments on mitral cells contained the following: 130 mM Cs-methanesulfonate, 4 mM NaCl, 0.5 mM CaCl_2_, 5 mM EGTA, 10 mM HEPES, 4 mM MgATP, 0.3 mM Na_2_GTP, and 5 mM QX314, with a pH of 7.35.

Electrophysiological signals were amplified using a MultiClamp 700B amplifier controlled via pClamp 10 software (Molecular Devices/Molecular Devices). Signals were acquired at 20 kHz via a Digidata 1440 analog/digital converter (Molecular Devices/Molecular Devices). Signals were filtered at 10 kHz at acquisition until processing with custom software written in MATLAB (see below, Data analysis). Access resistance was monitored at the beginning and end of each experiment, and recordings during which the access resistance increased by >50% or exceeded 40 MΩ were excluded from further analysis. Series resistance was not compensated.

For interneuron morphologic reconstructions, Alexa Fluor 488 hydrazide (100–200 µM, ThermoFisher/Invitrogen) was included in the recording solution. In these experiments, slices were mounted on a Acerra two-photon microscope (ThorLabs) and filled for 20–30 min. Morphologies were reconstructed from 100- to 300-µm optical stacks (1–2 µm/optical section) after performing manual morphologic tracing using the simple neurite tracer plugin in ImageJ (Longair et al., 2011).

### Data analysis

To assess intrinsic electrophysiological features, we subjected patched AOB neurons to a series of current clamp and voltage clamp challenges. Immediately after achieving the whole cell configuration, each cell’s resting membrane potential (V_rest_) was measured in current clamp mode. To standardize measurements across cells with different V_rest_, we injected steady-state currents to maintain each cell’s membrane potential (V_m_) between –70 and –75 mV. Based on initial measurements of input resistance (R_input_), we empirically determined the amplitude of hyperpolarizing current that adjusted V_m_ by approximately –50 mV (within the range of –120 to –125 mV). After determining this initial current injection amplitude, we generated a cell-specific 10-sweep Clampex protocol that applied increasingly depolarizing 0.5-s square current pulses, starting with the initial injection amplitude. For example, if the initial current injection was determined to be –100 pA, the 10-sweep protocol would have current injection increments of +20 pA (i.e., –100 pA, –80 pA, –60 pA,…,+80 pA). If the initial depolarization was determined to be –125 pA, the protocol would include increments of +25 pA, etc. This strategy allowed us to objectively challenge cells with widely varying V_rest_ and R_input_. In voltage clamp, cells were initially held at –70 mV, and a series of 12 voltage command steps (0.5 s in duration) were applied that spanned –100 to +10 mV.

For each cell, both current clamp and voltage clamp protocols were applied up to four times, and all reported quantities represent the mean responses across repeated trials. Twenty-six specific intrinsic parameters were extracted from each cell using custom software written in MATLAB. A description of these parameters in Figure 7*A* and the formulas used to calculate them is presented in Table 1. Values were normalized by the 95th percentile absolute value for each physiologic feature across the entire cell population (*N* = 150 cells). After normalization, resulting values were truncated to the range of –1 to 1. We performed cluster analysis on normalized values using a custom algorithm based on the mean-shift strategy (Comaniciu and Meer, 2002). Because of the relatively low dimensionality of descriptors and variable number of cells in each clustering run (i.e., when comparing IGCs and MCs only, versus all cells), we used bootstrapping to quantify the propensity for cells to co-cluster across statistical thresholds and subsets of parameter space, similar to (Meeks et al., 2010; Hammen et al., 2014). The propensity to co-cluster was quantified in a pairwise similarity matrix, which was used as the basis for final cluster assignment using the same mean-shift-based algorithm. Cluster assignments were manually inspected using heat map representations and multidimensional scaling plots to identify clustering conditions in which interpretable clusters were identified. Using this approach, we were able to use a single statistical threshold defining cluster boundaries (2.75 SD) for all clustering runs. Because each run analyzed a different number of cells, higher granularity was achieved in larger comparison sets.

**Table 1.** Parameters used for multidimensional analysis

Shorthand	Description	Mode	Method	Reference
V_rest_	Resting membrane potential	I Clamp	Direct measurement	N/A
Cm	Membrane capacitance	I Clamp	See reference	Golowasch et al. (2009)
Rm	Input resistance			
Ncomps	Number of model compartments			
Ih_sag	Hyperpolarization-induced depolarizing potential	I Clamp	$I_{H,sag}=V_{init}-V_{ss}$	N/A
Ih_current	Hyperpolarization-induced depolarizing current	V Clamp	$I_{H,curr}=I_{init}-I_{ss}$	N/A
EPSC_freq	Mean sEPSC frequency	V Clamp	Direct measurement after waveform sorting	Hendrickson et al. (2008)
EPSC_amp	Mean sEPSC amplitude	V Clamp		
EPSC_tau	Mean sEPSC decay constant	V Clamp	Least-squares exponential decay	N/A
S_freq_1	Spiking frequency, Lv. 1	I Clamp	Direct measurement	N/A
S_freq_2	Spiking frequency, Lv. 2	I Clamp	Direct measurement	N/A
S_freq_3	Spiking frequency, Lv. 3	I Clamp	Direct measurement	N/A
S_freq_4	Spiking frequency, Lv. 4	I Clamp	Direct measurement	N/A
S1_slope	Initial action potential rising slope	I Clamp	Initial spike derivative peak	Meeks et al. (2005)
S1_thresh	Initial action potential threshold	I Clamp	V_m_ at 10% of initial spike derivative	
S_accom_1	Spike rate accommodation, Lv. 1	I Clamp	$\frac{S_{init}-S_{final}}{S_{init}+S_{final}}$	N/A
S_accom_2	Spike rate accommodation, Lv. 2	I Clamp		
S_accom_3	Spike rate accommodation, Lv. 3	I Clamp		
S_accom_4	Spike rate accommodation, Lv. 4	I Clamp		
Na_curr_1	Voltage-gated Na^+^ current amplitude, Lv. 1	V Clamp	Direct measurement	N/A
Na_curr_2	Voltage-gated Na^+^ current amplitude, Lv. 2	V Clamp	Direct measurement	N/A
Na_curr_3	Voltage-gated Na^+^ current amplitude, Lv. 3	V Clamp	Direct measurement	N/A
Na_curr_4	Voltage-gated Na^+^ current amplitude, Lv. 4	V Clamp	Direct measurement	N/A
Na_curr_5	Voltage-gated Na^+^ current amplitude, Lv. 5	V Clamp	Direct measurement	N/A
K_curr_max	Voltage-gated K^+^ current, maximum	V Clamp	Direct measurement	N/A
K_curr_diff	Non-inactivating voltage-gated K^+^ current	V Clamp	Direct measurement	N/A

EPSCs were automatically detected and later separated from noise using a custom computer assisted wave form-based event sorting program written in MATLAB (Hendrickson et al., 2008). EPSC decay was measured by calculating the best fit single exponential for the decay period of the EPSC. Initial action potential rising slope was calculated by measuring the peak of the first derivative of voltage with respect to time (dV/dt). Threshold was defined as V_m_ at the time the dV/dt voltage reached 10% of its peak value. Membrane capacitance and input resistance were calculated according to current clamp-based multi-compartmental algorithms (Golowasch et al., 2009). Briefly, the voltage response of each cell to a hyperpolarizing current step was fit with a series of multi-exponential curves, and the best fit determined by identifying the solution with the lowest value of the Bayesian Information Criterion to avoid overfitting.

## Results

### Transcriptional diversity among AOB interneuron populations

**Figure 1.**
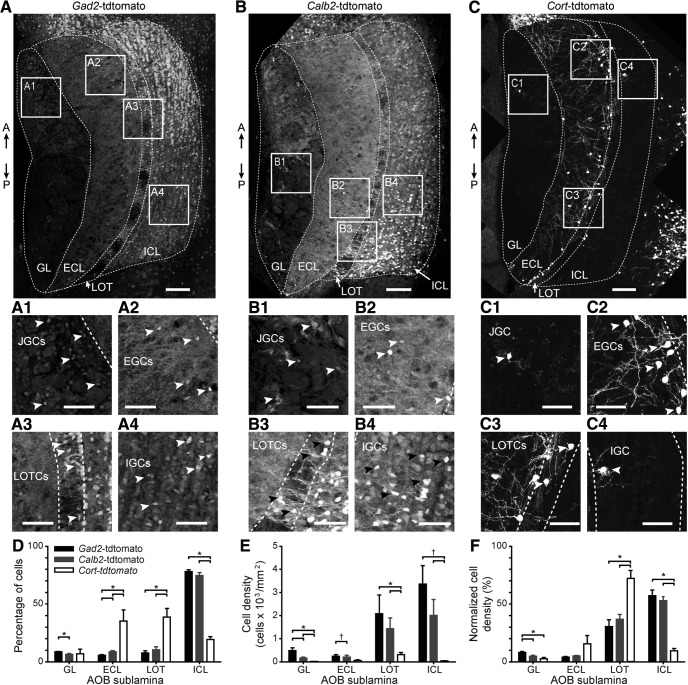
Cre-mediated genetic labeling of interneurons in all 4 AOB sublaminae. ***A***, Fluorescence micrograph of an adult male mouse AOB from a *Gad2*-tdTomato double transgenic mouse (*Gad2-*IRES*-*cre± x Rosa26-loxP-STOP-loxP-tdTomato±). Boxes labeled ***A1–A4*** correspond to the magnified inset panels below. A↑: anterior. P↓: posterior. ***A1–A4***, Magnified views of boxes from ***A*** centered on the GL, highlighting JGCs (***A1***), ECL, highlighting EGCs (***A2***), LOT, highlighting LOTCs (***A3***), and ICL, highlighting IGCs (***A4***). Arrowheads indicate the locations of selected tdTomato-positive interneuron cell bodies. ***B***, Fluorescence micrograph of an adult male *Calb2*-tdTomato double transgenic mouse. ***B1–B4***, Magnified views of boxes indicated in ***B***. ***C***, Fluorescence micrograph of an adult male *Cort*-tdTomato double transgenic mouse. ***C1–C4***, Magnified views of boxes indicated in ***C***. ***D***, Quantification of the percentage of all tdTomato-positive cells in the GL, ECL, LOT, and ICL from *Gad2*-tdTomato (black bars), *Calb2*-tdTomato (gray bars), and *Cort*-tdTomato mice (white bars). ***E***, Quantification of the cell density of tdTomato-positive cells by layer and genotype. ***F***, Quantification of the normalized cell density of tdTomato-positive cells by layer and genotype (normalization was within genotype). Scale bars in ***A–C***: 100 µm. Scale bars in magnified panels: 50 µm; *p < 0.05 and †0.05 < *p* < 0.1 (Student’s unpaired, two-tailed *t* test).

Several electrophysiological studies have shown that the AOB is organized quite differently than the adjacent, and superficially similar, MOB (Araneda and Firestein, 2006; Smith et al., 2009; Leszkowicz et al., 2012; Shpak et al., 2012; Taniguchi et al., 2013). Interneuron types in the MOB have been identified based on the selective expression of marker genes (Parrish-Aungst et al., 2007; Kato et al., 2013; Miyamichi et al., 2013). In the AOB, interneuron classifications have been made based on antibody staining for markers including calbindin (*Calb1* gene; Porteros et al., 1995), calretinin (*Calb2* gene; Jacobowitz and Winsky, 1991), GABA (Takami et al., 1992), and somatostatin (*Sst* gene; Takami et al., 1990). With the goal of studying the functional diversity among morphologically and genetically labeled AOB interneuron types, we screened through several transgenic mouse lines in which AOB interneurons could be labeled via cre-mediated genetic recombination (Fig. 1*A–C*). We identified three promising lines in which strong cre-mediated gene expression was observed in AOB interneurons: *Gad2-cre*, *Calb2-cre*, and *Cort-cre* (Taniguchi et al., 2011). In each line, cre expression was introduced via knock-in to the 3’ untranslated region of the gene, leaving the endogenous coding regions intact. *Cre* transgenic mice were mated to *Rosa26-loxP-STOP-loxP-tdTomato* reporter mice (“Ai9” strain; Madisen et al., 2010) to produce transgenic mice in which specific populations of AOB interneurons were fluorescently labeled (referred to as *Gad2*-tdTomato, *Calb2*-tdTomato, and *Cort*-tdTomato animals; Fig. 1*A–C*).

We observed strong tdTomato expression in all AOB cell layers in adult *Gad2*-tdTomato and *Calb2*-tdTomato mice (Fig. 1*A*,*B*), consistent with antibody staining observations (Jacobowitz and Winsky, 1991; Takami et al., 1992; Parrish-Aungst et al., 2007). The percentage of both *Gad2*-tdTomato-positive and *Calb2*-tdTomato-positive cells was greatest in the internal cellular layer (ICL) of the AOB, which contains neurons called internal granule cells (IGCs), the most plentiful neuronal type in the AOB (*N* = 4; Fig. 1*D*). The overall density of *Gad2*-tdTomato+ and *Calb2*-tdTomato+ neurons was greatest in the ICL and adjacent lateral olfactory tract (LOT; *N* = 4; Fig. 1*E*). *Gad2*-tdTomato+ and *Calb2*-tdTomato+ populations, despite densely labeling IGCs, appeared to label partially non-overlapping populations. The pattern of *Gad2*-tdTomato+ and *Calb2*-tdTomato+ IGCs varied along the anterior-posterior axis of the AOB, with *Gad2*-tdTomato+ IGCs enriched in the anterior AOB (aAOB) and *Calb2*-tdTomato+ IGCs enriched in the posterior AOB (pAOB; Fig. 1*A*,*B*). These anterior-posterior biases were most prominent in the lateral-most regions of the AOB (*N* = 3).

In contrast to the broad *Gad2*-tdTomato and *Calb2*-tdTomato labeling, we observed highly selective labeling of EGCs, a recently described AOB interneuron type, in *Cort*-tdTomato double transgenic mice (Larriva-Sahd, 2008; Fig. 1*C*). EGCs are multi-polar cells that possess elaborate, spine-laden dendrites and, like IGCs, lack an apparent axon (Larriva-Sahd, 2008). The percentage of *Cort*-tdTomato+ interneurons was greatest in the ECL and adjacent LOT, but these neurons were scarce in the glomerular layer (GL) and ICL (*N* = 4; Fig. 1*D*). We also noted a dense cluster of *Cort*-tdTomato+ neurons in the MOB at the anterior/dorsal border between the AOB and MOB, but because they were outside the AOB proper we did not subject them to further analysis here. The overall density of *Cort*-tdTomato labeling was much lower than *Gad2*- or *Calb2*-tdTomato populations (*N* = 4; Fig. 1*E*). The percentage of *Cort*-tdTomato+ cells, when normalized by laminar area (normalized cell density; Fig. 1*F*), revealed that these cells were tightly clustered along the border of the ECL and LOT (*N* = 4). These three transgenic driver lines thus label interneurons in all AOB sublaminae, and suggested transcriptional diversity even within morphologic types.

### Electrophysiological characteristics of AOB IGCs

IGCs are the predominant neuronal type in the AOB, and have been studied more thoroughly than any other interneuron in this circuit (Jia et al., 1999; Taniguchi and Kaba, 2001; Araneda and Firestein, 2006; Smith et al., 2009, 2015; Taniguchi et al., 2013; Zimnik et al., 2013; Hu et al., 2016). These interneurons are thought to play prominent roles in AOB function, including plasticity (Bruce, 1959; Kaba and Keverne, 1988; Brennan et al., 1990; Okutani et al., 2002; Araneda and Firestein, 2006). Despite the likely importance of AOB IGCs for AOB computations, their electrophysiological features have not been extensively studied. Given the transcriptional diversity within the IGC population (Fig. 1), we hypothesized that IGCs, as a class, comprise multiple, functionally distinct subpopulations. We further hypothesized that type distinctions may be identified by thoroughly evaluating IGC intrinsic physiologic features, a strategy that has been successful in other neural circuits (Cadwell et al., 2017; Tavakoli et al., 2018; Ting et al., 2018).

**Figure 2.**
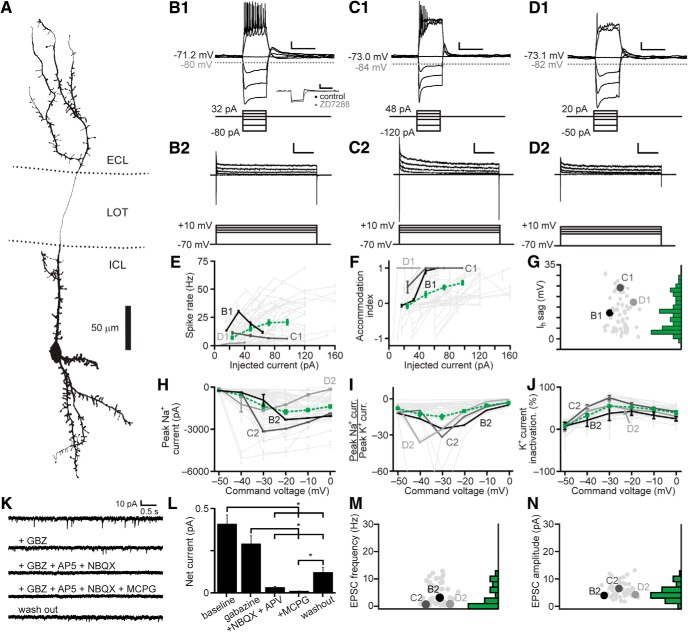
Electrophysiological properties of AOB IGCs. ***A***, Morphologic reconstruction of an IGC dendritic arbor. ***B1–D1***, Responses of three representative IGCs (top) to current clamp challenges (bottom). Scale bars: 10 mV, 500 ms. Inset in ***B1*** shows the blockade of the hyperpolarization-activated depolarizing I_H_ sag potential by ZD7288 (10 µM). ***B2–D2***, Responses of the same three IGCs (top) to a series of command potential steps from –70 mV in voltage clamp (bottom). Hyperpolarizing responses were also recorded, but are not shown. Scale bars: 500 pA, 100 ms. ***E***, Spike rate input-output curves for IGCs subjected to a series of step depolarizations in current clamp. Dashed green line indicates the mean ± SE (*N* = 52). ***F***, Spike accommodation index for IGCs subjected to the same series of step depolarizations shown in ***C*** (*N* = 52). ***G***, Maximal I_H_ sag potential for 52 IGCs. ***H***, Input-output curves for peak inward voltage-gated Na^+^ currents (*N* = 51). ***I***, Input-output curves for the ratio of peak voltage-gated Na^+^ to peak voltage-gated K^+^ currents (*N* = 52). ***J***, Input-output curve for normalized voltage-gated K^+^ current inactivation (*N* = 52). ***K***, Representative recordings of spontaneous synaptic currents (command potential –70 mV) before and during blockade of GABA_A_ receptors with gabazine (2.5 µM), AMPA and NMDA receptors (10 µM AP5, 1 µM NBQX), and Type I/II mGluRs (100 µM MCPG, *N* = 15). ***L***, Blockade of net spontaneous synaptic currents during pharmacological blockade (holding potential –70 mV). Asterisks indicate *p* < 0.05 by multiple comparisons of mean ranks, Kruskal–Wallis test. ***M***, sEPSC frequency (*N* = 52). ***N***, sEPSC amplitude (*N* = 52).

We developed an unbiased assay involving a series of electrophysiological challenges in whole cell current clamp and voltage clamp, and applied these challenges to 58 IGCs of multiple transcriptional types (Fig. 2). This approach revealed several consistent features among IGCs, most notably a propensity for spike accommodation on moderate somatic depolarization (Fig. 2*B–F*) and a prominent HCN-channel mediated I_H_ “sag” potential that was blocked by the HCN channel antagonist ZD7288 (10 µM; Fig. 2*B–D*,*G*). IGCs displayed moderate rates of spontaneous EPSCs (sEPSCs) mediated by AMPA-type and NMDA-type ionotropic glutamate receptors and metabotropic glutamate receptors (Fig. 2*K–N*). Despite these relatively consistent features, we observed remarkable intrinsic feature variability among IGCs (Fig. 2*E–J*), consistent with the hypothesis that the IGC class may comprise several distinct subpopulations.

**Figure 3.**
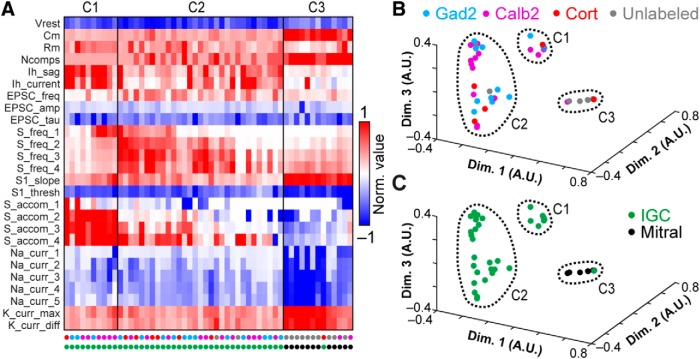
Multidimensional analysis of IGC physiologic properties. ***A***, Multidimensional analysis of 26 intrinsic physiologic properties was used to investigate potential relationships between different genetically defined populations of IGCs, with mitral cells included for comparison. Each column represents the physiologic profile of a single cell. Data were normalized to the 95th percentile absolute value observed for each feature across 150 AOB neurons, including IGCs, EGCs, JGCs, and mitral cells. 42/58 recorded IGCs and 12/17 mitral cells contained information for all 26 parameters and were subjected to cluster analysis. Row labels refer to the intrinsic properties listed in Table 1, and markers below each column are color coded based on genetically defined and morphologically defined type (as in ***B***, ***C***). Clusters are separated by the vertical lines. ***B***, ***C***, Nonclassical multidimensional scaling analysis of the 54 cells shown in ***A***. Each symbol represents a single cell. Relative symbol positions reflect similarity across all 26 measurements (dimensions). In ***B***, colored symbols indicate the genetic type of the recorded neurons; in ***C***, colored symbols indicate the morphologic type (same color scheme as ***A***). Dashed lines identify each cluster from ***A***.

To test whether the variability in electrophysiological features might be associated with the different genetic/transcriptional labels (Fig. 1), we performed multidimensional analysis on a panel of 26 objectively measured intrinsic features (Cansler et al., 2017; Table 1; Fig. 3). In this analysis we included recordings from 12 AOB MCs that had undergone the same battery of intrinsic physiologic challenges. To account for the considerable variation in these physiologic properties across cell types, data for each parameter were normalized by the 95th percentile absolute value across all 150 recordings in this study, then truncated to fit within the range of –1 to 1 (see Materials and Methods). Analysis of a population of 42 IGCs and 12 MCs from which we had successfully extracted all 26 parameters revealed three macroscopic clusters, two of which exclusively contained IGCs, and the other of which was dominated by MCs (Fig. 3*A*). Sorting the cells within each cluster using principal components analysis (PCA) further revealed diversity within one of the two IGC clusters (Fig. 3*A*). This division was made clearer by performing multidimensional scaling of the matrix subjected to final clustering (Fig. 3*B–C*). The first cluster contained a large cohort of IGCs that demonstrated strong I_H_ conductances, fired high frequency bursts of action potentials, but failed to sustain high rates of firing in the face of moderate somatic depolarization (Fig. 3*A*). The second cluster was more distributed (visible in the multidimensional scaling analysis in Fig. 3*B*,*C*). The first subgroup within the second IGC cluster (Fig. 3*A*) contained IGCs that were better capable of sustaining high frequency spiking without strong accommodation and relatively low I_H_ conductances. The other subgroup within Cluster 2 contained cells that were poorly able to spike even with moderate to strong somatic depolarization, but possessed moderate I_H_ conductances and showed clear evidence of spontaneous glutamatergic input. The final cluster was dominated by mitral cells, which were clearly distinguishable from IGCs through large differences in several properties (Fig. 3*A–C*). The likely reason that the sub-clusters within Cluster 2 were not automatically identified by this clustering algorithm, which identifies cluster boundaries based on multidimensional statistical variance (Comaniciu and Meer, 2002; Turaga and Holy, 2012), is that this dataset had relatively large variance and low total number of cells.

Having defined several subgroups among IGCs based on this multidimensional approach, we proceeded to test whether any of these clusters showed evidence of being enriched in any of the 3 transgenic lines used to label IGCs (Fig. 1). A binomial test for differential inclusion in the two IGC clusters based on genotype indicated only one such association, which was moderate enrichment of *Calb2*-tdTomato IGCs in Cluster 1 (6/10 cells, base probability 35.1%, *p* = 0.027). By contrast, this same analysis showed clear evidence for enrichment of mitral cells in Cluster 3 (12/13 cells, base probability 22.2%, *p* = 3.2 × 10^−9^). Multidimensional scaling produced no further support for the hypothesis that intrinsic physiologic differences within the IGC population were strongly associated with a particular transgenic label. These results suggest that IGCs are both transcriptionally and physiologically diverse, but that these particular genetic labels do not strongly correlate with intrinsic physiologic features.

### Electrophysiological characteristics of AOB EGCs

In the MOB, parvalbumin-expressing interneurons in the external plexiform layer perform MC gain scaling (Kato et al., 2013; Miyamichi et al., 2013). The AOB lacks these parvalbumin-expressing interneurons (data not shown), but possesses transcriptionally diverse set of EGCs (*Gad2*, *Calb2*, and *Cort* expressing; Fig. 1). We hypothesized that EGCs, which possess broad, densely spine-laden dendrites (Fig. 1; Larriva-Sahd, 2008), would possess electrophysiological properties that are distinct from IGCs, and which may vary by genetically labeled type.

**Figure 4.**
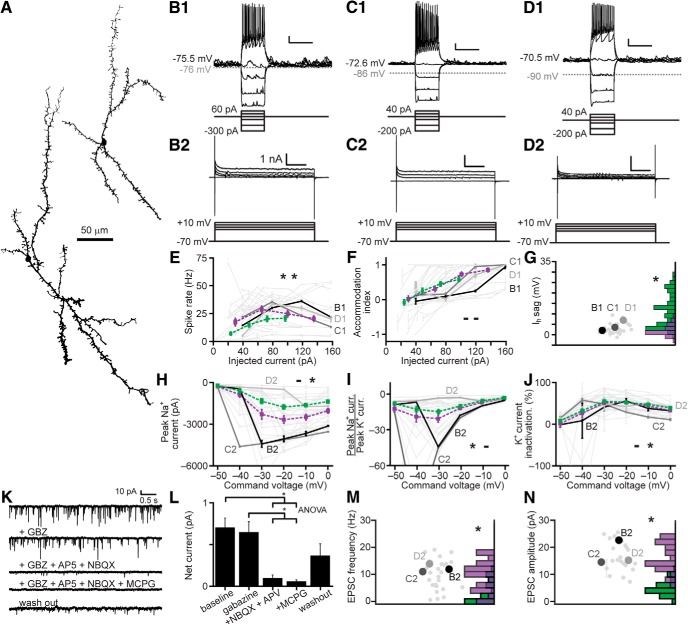
Electrophysiological properties of AOB EGCs. ***A***, EGC morphologic reconstructions. ***B1****–****D1***, Responses of three representative EGCs to current clamp challenges. Scale bars: 10 mV, 500 ms. ***B2–D2***, Responses of the same 3 EGCs to a series of command potential steps from –70 mV in voltage clamp. Hyperpolarizing responses were also recorded, but are not shown here. Scale bars: 1 nA, 100 ms. ***E***, Spike rate input-output curves for EGCs. Dashed purple line indicates the mean ± SE for EGCs, and dashed green for IGCs (from Fig. 2*E*; *N* for EGCs = 33, two-way ANOVA compared to IGCs: *p* = 1.48 × 10^−5^ main effect of cell type, *p* = 0.0051 interaction between cell type and stimulus intensity). ***F***, Spike accommodation index for EGCs (*N* = 33, two-way ANOVA compared to IGCs: *p* = 0.83 main effect of cell type. *p* = 0.113 interaction between cell type and stimulus intensity). ***G***, Maximal I_H_ sag potential (*N* = 33, compared to IGCs *p* = 1.48 × 10^−9^ by Wilcoxon rank-sum test). ***H***, Input-output curves for peak inward voltage-gated Na^+^ currents (*N* = 29, two-way ANOVA compared to IGCs: *p* = 0.518 main effect of cell type, *p* = 0.0267 interaction between cell type and stimulus intensity). ***I***, Input-output curves for the ratio of peak voltage-gated Na^+^ to peak voltage-gated K^+^ currents (*N* = 29, two-way ANOVA compared to IGCs: *p* = 0.0048 main effect of cell type, *p* = 0.0916 interaction between cell type and stimulus intensity). ***J***, Input-output curve for normalized voltage-gated K^+^ current inactivation (*N* = 29, two-way ANOVA compared to IGCs: *p* = 0.0381 main effect of cell type, *p* = 0.1823 interaction between cell type and stimulus intensity). ***K***, Representative recordings of spontaneous synaptic currents (command potential –70 mV) before and during application of gabazine, AP5, NBQX, and MCPG (*N* = 5). ***L***, Blockade of net spontaneous synaptic currents during pharmacological blockade (holding potential –70 mV). Asterisks indicate *p* < 0.05 by multiple comparisons of mean ranks, one-way ANOVA. ***M***, sEPSC frequency (*N* = 29, *p* = 2.84 × 10^−6^ Wilcoxon rank-sum test). Purple bars refer to EGCs, green bars to IGCs. ***N***, sEPSC amplitude (*N* = 29, *p* = 9.53 × 10^−13^ Wilcoxon rank-sum test).

We therefore measured the intrinsic and synaptic properties of EGCs using the same protocols used for IGCs (Fig. 4). Several differences between EGCs and IGCs were apparent (Fig. 4*B–D*). First, resting membrane potentials for EGCs were strongly hyperpolarized (–83.6 ± 1.3 mV, *N* = 31) compared to IGCs (–75.6 ± 1.3 mV, *N* = 31, *p* = 1.27 × 10^−4^ Wilcoxon rank-sum test). EGCs responded to depolarization with sustained 20- to 30-Hz spiking, showing accommodation only at the highest somatic current injections (Fig. 4*E*,*F*). EGC recordings revealed small I_H_ sag potentials that were dramatically smaller than those observed in IGCs (Fig. 4*G*). Consistent with the higher rates of sustained spiking in many EGCs, we observed stronger voltage-gated Na^+^ and K^+^ currents in EGCs than in IGCs (Fig. 4*H*,*I*). Despite this difference in K^+^ current magnitude, EGC K^+^ currents were similar to those of IGCs in their proportion of slow inactivation (Fig. 4*J*). Like IGCs, sEPSCs were overwhelmingly carried by AMPA- and NMDA-type glutamate receptors (Fig. 4*L*), but the frequency and amplitudes of sEPSCs onto EGCs were many-fold higher than those of IGCs, indicating much denser connectivity with MCs on a per-cell basis (Fig. 4*M*,*N*).

**Figure 5.**
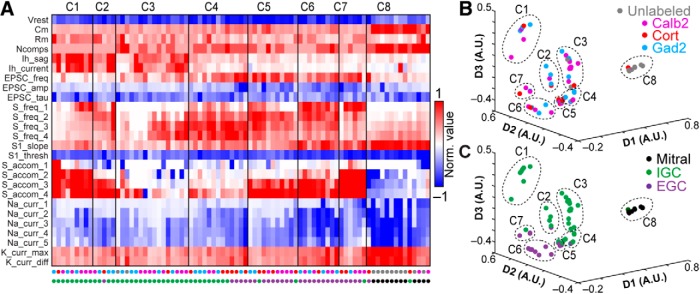
Multidimensional analysis of IGC and EGC physiologic properties. ***A***, Cluster analysis of IGCs and EGCs with mitral cells included for comparison. Normalized values were calculated as per Figure 3. 29/35 recorded EGCs had information for all 26 parameters. EGC data were combined with the population of neurons in Figure 3 and re-clustered. Row labels refer to intrinsic properties listed in Table 1. Each column represents a single cell, and column labels are color coded based on genetically defined type and morphologically defined type. Clusters are separated by solid vertical lines. ***B***, ***C***, Nonclassical multidimensional scaling of the 83 cells shown in ***A***, colorized by genetically defined type (***B***) and morphologically defined type (***C***). Dashed outlines indicate approximate cluster boundaries. ***C1–C8*** refer to the cluster definitions in ***A***.

The readily observable differences in intrinsic features suggested that EGCs might be distinguishable from IGCs when all physiologic properties were considered. We performed multidimensional clustering across IGCs and EGCs by combining the physiologic profiles of 29 EGCs to the group of 42 IGCs and 12 MCs analyzed in Figure 3 and re-clustering the data (Fig. 5). This analysis revealed the presence of 8 clusters within the combined dataset. Consistent with our hypothesis, Clusters 1 and 3 were overwhelmingly comprised of IGCs, Cluster 4 included fast-spiking IGCs and some EGCs, Clusters 5–7 were overwhelmingly comprised of EGCs, and Cluster 8 was dominated by MCs (Fig. 3*A*). As was the case with IGCs, we found little evidence for strong associations between these physiologically derived clusters and transcriptional type (3/5 *Gad2*-tdTomato cells in Cluster 2, *p* = 0.022; 6/13 cells in Cluster 4, *p* = 0.011). On the other hand, enrichment based on morphologic type was clear in all clusters except Cluster 4. These results further support the observation that EGC intrinsic physiologic features are clearly distinct from IGCs, but that these features do not correlate with labeling via these three transgenic strategies.

### Electrophysiological characteristics of AOB JGCs

**Figure 6.**
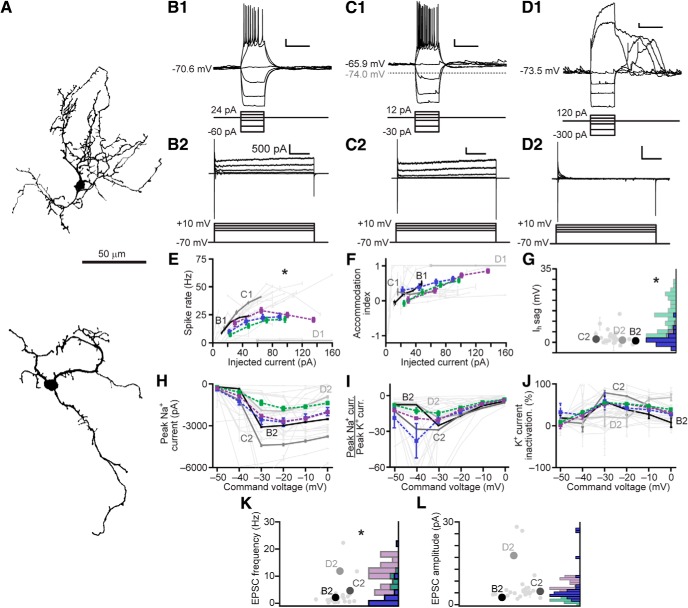
Electrophysiological properties of AOB JGCs. ***A***, JGC morphologic reconstructions. ***B1****–****D1***, Responses of three representative JGCs (top) to current clamp challenges (bottom). Scale bars: 10 mV, 500 ms. ***B2–D2***, Responses of the same three JGCs (top) to a series of command potential steps from –70 mV in voltage clamp (bottom). Hyperpolarizing responses were also recorded, but are not shown here. Scale bars: 500 pA, 100 ms. ***E***, Spike rate input-output curves for JGCs. Dashed red line indicates the mean ± SE for JGCs, dashed gray for IGCs, dashed black from EGCs (*N* = 27; two-way ANOVA compared to IGCs: *p* = 0.414 main effect of cell type, *p* = 0.850 interaction between cell type and stimulus intensity; two-way ANOVA compared to EGCs: *p* = 0.032 main effect of cell type, *p* = 0.0254 interaction between cell type and stimulus intensity). ***F***, Spike accommodation index for JGCs (*N* = 27, two-way ANOVA compared to IGCs: *p* = 0.013 main effect of cell type. *p* = 0.078 interaction between cell type and stimulus intensity; two-way ANOVA compared to EGCS: *p* = 0.041 main effect of cell type, *p* = 0.007 interaction between cell type and stimulus intensity). ***G***, Maximal I_H_ sag potential (*N* = 30, compared to IGCs *p* = 1.07 × 10^−10^, to EGCs 0.07 by Wilcoxon rank-sum test). ***H***, Input-output curves for peak inward voltage-gated Na^+^ currents (*N* = 27, two-way ANOVA compared to IGCs: *p* = 0.065 main effect of cell type, *p* = 0.323 interaction between cell type and stimulus intensity; two-way ANOVA compared to EGCs: *p* = 0.346 main effect of cell type, 0.456 interaction between cell type and stimulus intensity). ***I***, Input-output curves for the ratio of peak voltage-gated Na^+^ to peak voltage-gated K^+^ currents (*N* = 28, two-way ANOVA compared to IGCs: *p* = 0.0001 main effect of cell type, *p* = 0.0059 interaction between cell type and stimulus intensity; two-way ANOVA compared to EGCs: *p* = 0.052 main effect of cell type, *p* = 0.148 interaction between cell type and stimulus intensity). ***J***, Input-output curve for normalized voltage-gated K^+^ current inactivation (*N* = 28, two-way ANOVA compared to IGCs: *p* = 0.0001 main effect of cell type, *p* = 0.001 interaction between cell type and stimulus intensity; two-way ANOVA compared to EGCs: *p* = 0.054 main effect of cell type, *p* = 0.0752 interaction between cell type and stimulus intensity). ***K***, sEPSC frequency (*N* = 27, *p* = 0.21 to IGCs *p* = 4.77 × 10^−6^ to EGCs by Wilcoxon rank-sum test). ***L***, sEPSC amplitude (*N* = 27, *p* = 2.61 × 10^−8^ to IGCs, *p* = 6.10 × 10^−4^ to EGCs by Wilcoxon rank-sum test).

The MOB has several interneuron types, collectively termed JGCs, which reside in and around the GL (Larriva-Sahd, 2008; Yokosuka, 2012). In the MOB, JGCs include excitatory external tufted cells, several types of inhibitory periglomerular cells (PGs), and short axon cells (SAs; Shipley and Ennis, 1996; Schoppa et al., 1998; Aungst et al., 2003; Parrish-Aungst et al., 2007; Shao et al., 2009; Liu et al., 2013; Najac et al., 2015; Burton et al., 2017; Geramita and Urban, 2017). In the AOB, very few electrophysiological inquiries have been undertaken on JGCs (Goldmakher and Moss, 2000; Hu et al., 2016). We performed electrophysiological recordings from JGCs in *Gad2*-tdTomato, *Calb2*-tdTomato, and *Cort*-tdTomato transgenic mice, using the electrophysiological profiling strategy used for IGCs and EGCs (Fig. 6).

We observed two distinct morphologic and electrophysiological types in the AOB GL, potentially including PGs and some SAs (Fig. 6*A–D*). Some AOB JGCs had low capacitance and responded to moderate depolarization with sustained action potential generation (Fig. 6*B*,*C*). These JGCs responded to depolarizing voltage steps with non-accommodating voltage-gated K^+^ currents, and often showed a slowly activating K^+^ current (Fig. 6*B*,*C*). Other AOB PGs, by contrast, had higher capacitance and lower input resistance, suggesting a larger and/or more highly conductive dendritic arbor. These cells often responded to mild depolarization by generating plateau potentials (Fig. 6*D*). Plateau potentials in these cells were sometimes observed in the absence of somatic current injection, or following hyperpolarizing current injections, suggesting that sEPSPs and/or rebound depolarization were sufficient to drive these events. Unlike regular-spiking JGCs, plateau potential-generating JGCs responded to voltage-clamp depolarization with nearly completely inactivating K^+^ currents (Fig. 6*D*). JGCs, taken as a whole, demonstrated mean physiologic features that between IGCs and slightly lower than EGCs (Fig. 6*E–L*). This was likely a by-product of the apparent split between putative PGs and SAs.

**Figure 7.**
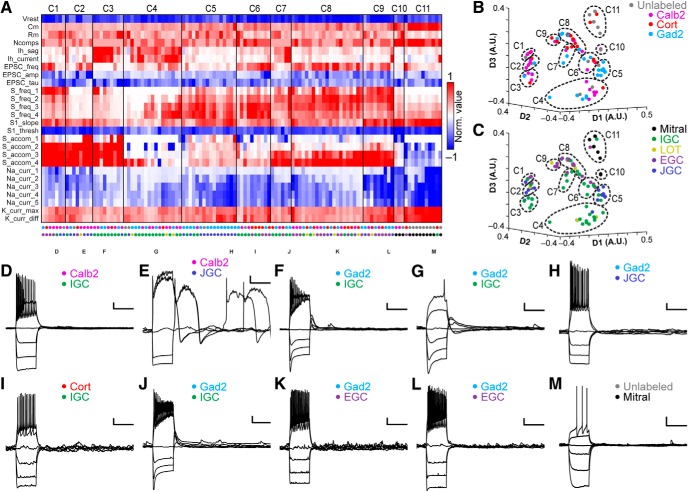
Multidimensional analysis across AOB neuronal types. ***A***, Cluster analysis of the 118 interneurons and mitral cells for which all 26 physiologic properties were obtained. Normalized values were calculated as per Figure 3. Row labels refer to intrinsic properties listed in Table 1. Each column represents a single cell, and column labels are color coded based on genetically defined type and morphologically defined type. Clusters are separated by solid vertical lines. ***B***, ***C***, Nonclassical multidimensional scaling of the cell properties shown in ***A***, colorized by genetically defined type (***B***) and morphologically defined type (***C***). Dashed outlines indicate approximate cluster boundaries. ***C1–C11*** refer to the cluster definitions in ***A***. ***D–M***, Current clamp responses to the same current injection series shown in Figures 2, 4, 6 from selected cells as noted below the columns in ***A***. Scale bars: 10 mV, 500 ms.

To evaluate the collective physiologic differences between AOB JGCs, IGCs, and EGCs, and potentially associated with genetically labeled types, we introduced the physiologic features of 26 JGCs and nine LOT cells (LOTCs) into the dataset containing IGCs, EGCs, and MCs (total 118 neurons) and re-clustered the data. This analysis identified 11 clusters and a spectrum of electrophysiological properties that spanned the diverse set of AOB interneurons (Fig. 7). As in previous clustering analyses, subdivisions within the JGC population became evident, with plateau potential-generating and regular-spiking JGCs associated with different clusters (Clusters 2 and 5, respectively; Fig. 7*A–C*). Cluster 1 from this analysis contained faster-spiking, but strongly accommodating cells lacking prominent I_H_ currents (four EGCs, one IGC, and two JGCs; Fig. 7*D*). Cluster 2 contained cells that spiked extremely weakly and showed rapid spike adaptation, and was enriched in plateau potential-generating JGCs (6/9 cells in cluster), all of which were labeled in *Calb2*-tdTomato transgenic lines (Fig. 7*E*). Clusters 3 and 4 were enriched in weakly spiking IGCs (8/9 in Cluster 3; 13/17 in Cluster 4; Fig. 7*F*,*G*). Cluster 5 was enriched in regular-spiking JGCs (13/16 cells in cluster), all of which were labeled in *Gad2*-tdTomato transgenic mice (Fig. 7*H*). Thus, multidimensional analysis confirmed the apparent split of JGCs into regular-spiking and plateau potential-generating subgroups, and further revealed that the *Gad2*-tdTomato-labeled JGCs were enriched in the regular-spiking phenotype, while *Calb2*-tdTomato-labeled JGCs were enriched in the plateau potential-generating phenotype. Clusters 6 and 7, similarly to Cluster 1, included a mix of faster-spiking IGCs and EGCs (Fig. 7*I*,*J*). Clusters 8 and 9 were enriched in EGCs of all transcriptional types (12/21 in Cluster 8; 6/9 in Cluster 9) but also included a substantial number of LOTCs (5/30 across the two clusters; Fig. 7*K*,*L*). The apparent split among LOTCs into IGC/JGC-dominated clusters (4/9 LOTCs in Clusters 2–4) and EGC-dominated clusters (5/9 LOTCs in Clusters 8–9) indicates that LOTCs include a mix of IGC-like neurons and EGC-like neurons. As in other clustering runs, Clusters 10 and 11 were dominated by MCs (12/14 cells across the two clusters). Taken as a whole, clustering based on this set of intrinsic electrophysiological parameters provided useful information that identifies functional subdivisions within morphologic classes and an objective mechanism for evaluating relationships between genetically defined and morphologically defined cell types.

## Discussion

### Transcriptional diversity among AOB interneurons is largely uncorrelated with intrinsic physiology

A major practical limitation to studying information processing in neural circuits is the available toolkit with which to selectively manipulate circuit elements. These tools can include electrical, optical, or pharmacological manipulations that selectively engage circuit elements at a particular point in space and/or time, and are increasingly involving conditional expression systems that leverage cell type-specific gene expression patterns. In a quest to improve on our toolkit for studying the neural circuit logic of the AOB, we designed the studies presented here to be capable of objectively distinguishing between physiologically distinct cell types within and across morphologic and transcriptional categories. This general strategy is becoming increasingly important as novel technologies for combining electrophysiological and transcriptional data at the single cell level (Chen et al., 2016; Fuzik et al., 2016; Cadwell et al., 2017; Ting et al., 2018).

Taken as a whole, the results of these studies highlight the current challenges to circuit-level inquiries in understudied brain regions. Despite successfully identifying several physiologically distinct interneuron subpopulations in the AOB (especially within the morphologically defined classes of IGCs and JGCs; Figs. 2, 3, 6, 7), in only one case did we observe any specific segregation of genetic/transcriptional labels based on within-morphologic-class physiologic features (JGCs; Figs. 6, 7). That said, the *Cort-cre* transgenic line, which labels interneurons sparsely throughout the brain, was almost fully selective for EGCs in the AOB (Fig. 1). The *Cort-cre* transgenic mouse line thus provides a compelling genetic tool for selective monitoring or manipulation of a distinct subtype of AOB interneurons; we expect this tool to be highly useful for future studies of AOB EGC function. Even so, the *Cort*-tdTomato EGC population is not pure (there are a few *Cort*-tdTomato IGCs and PGCs; Figs. 1–7), and it is not clear whether every AOB EGC is labeled in the *Cort*-cre transgenic line. This survey of genetically labeled AOB interneurons revealed other differences in expression pattern, for example the varying densities of *Gad2*-tdTomato and *Calb2*-tdTomato in the ICL (Fig. 1*A*,*B*). These observations suggest the possibility of additional functional differences within these genetically labeled types that would require additional experiments to uncover. Ultimately, these studies indicate that new tools will be necessary for researchers to selectively monitor and manipulate functional groups of AOB interneurons. Such tools will almost certainly involve the identification of new marker genes, but may also involve the utilization of tools that selectively label interneurons based on recent activation (Cansler et al., 2017; Gao et al., 2017).

### IGC physiologic diversity suggests multiple functionally distinct subpopulations

IGCs are the most numerous and are currently the best-studied interneuron subtype in the AOB (Dudley and Moss, 1999; Jia et al., 1999; Araneda and Firestein, 2006; Smith et al., 2009; Taniguchi et al., 2013; Hu et al., 2016). Their known and hypothesized connections to experience-dependent plasticity make them a particularly compelling cell type to study (Brennan et al., 1990; Okutani et al., 1999; Cansler et al., 2017; Gao et al., 2017; Oboti et al., 2017). Despite the relatively strong set of inquiries into IGC function, there are many outstanding questions about IGCs’ role in AOB information processing and experience-dependent chemosensory plasticity. In this study, we did not focus on any specific aspect of IGC function, but instead explored the intrinsic physiologic diversity within the IGC subtype in hopes of providing an improved quantitative foundation for future AOB studies.

The results of our systematic inquiry into IGC intrinsic features revealed at least 2, if not more, functionally defined subpopulations of IGCs (Figs. 2, 3). The most prominent differences between these subpopulations were the capacity to fire sustain action potential trains in the face of moderate depolarization and the magnitude of their HCN channel-mediated I_H_ sag potentials (Figs. 2, 3). The functional implications of these results for AOB circuit function are not yet clear, but the data suggest, at a minimum, that AOB IGCs should not be considered to be a monolithic population. A substantial fraction of recorded AOB IGCs are poorly capable of generating more than one or two action potentials (Figs. 2*D*, 3). Given that AOB IGCs are replenished through adult-born neurons migrating through the rostral migratory stream (for review, see Oboti and Peretto, 2014), it is possible that these IGCs, which are not selectively labeled by any of the three transgenic lines used in this study, represent immature (i.e., recently arrived) adult-born neurons that have not yet fully incorporated into the AOB circuit. Even still, these IGCs exhibit sEPSCs of normal frequency and amplitude, suggesting they possess dendrites and functional synapses, and as such are theoretically capable of contributing to AOB processing even if they are “immature.” This feature, functional activity before full maturity, has also been seen in peripheral olfactory sensory neurons (Cheetham et al., 2016). Studying the conditions that bring about and contribute to neuronal maturation of IGCs seems likely to be an important component of inhibitory function in sensory processing (Oboti and Peretto, 2014).

Other functionally defined subpopulations of AOB IGCs include neurons that vary in their capacity to sustain high frequency spiking in the face of intermediate levels of somatic depolarization (Figs. 2, 3). The expression of the prominent HCN channel-mediated I_H_ conductance in cells displaying intermediate intrinsic excitability (i.e., neurons capable of firing high frequency bursts, but readily entering depolarization block; Figs. 2*C*, 3) may indicate an intermediate functional phenotype among IGCs. This hypothesis is partially supported by recent studies that showed IGC intrinsic physiology is altered over 2- to 8-h time courses by recent chemosensory-driven activity (Cansler et al., 2017; Gao et al., 2017). It is an intriguing possibility that these other, more subtle, differences in IGC physiology may reflect their involvement in recent activation. Given that AOB IGCs are hypothesized to contribute to experience-dependent chemosensory learning (Bruce and Parrott, 1960; Kaba and Keverne, 1988; Brennan et al., 1990; Okutani et al., 1999), it may be the case that the IGC physiologic profiles established in this study are capable of distinguishing IGCs that span the range of immature, to mature but quiescent, to actively engaged IGCs.

### EGC physiologic features suggest unique roles in AOB sensory processing

In the MOB and AOB, like most cortically arranged brain circuits, interneurons populate major plexiform layers. In the MOB, parvalbumin-expressing interneurons in the external plexiform layer (PV-EPL interneurons) have been shown to possess unique physiologic features that support divisive normalization (Kato et al., 2013; Miyamichi et al., 2013). Recent reports indicated a morphologically similar class of interneurons, EGCs, is present in the rodent AOB, and each of the transgenic lines used in this study labeled EGCs (Fig. 1). It is not yet clear whether AOB EGCs and MOB PV-EPL interneurons represent analogous cell types. For example, EGC morphology and physiology is also similar to MOB superficial granule cells (Geramita et al., 2016). Nevertheless, they share key physiologic features (and soma position) with MOB PV-EPL interneurons, including high frequency spontaneous excitatory synaptic input and relatively high sustained firing rates. AOB EGCs possess broad, spinous dendrites that spread out along the anterior-posterior axis of the AOB (Larriva-Sahd, 2008; Fig. 4). Based on morphology alone, one might hypothesize that these cells may support broad innervation and gain-scaling. Since there are no previous reports of AOB EGC physiology, we were excited to explore the physiologic features that, along with the morphologic features of these cells (Fig. 4), might inform hypotheses about their function.

The intrinsic physiology of AOB EGCs was unique and in some ways extreme (Figs. 4, 5). AOB EGCs possessed resting membrane potentials, averaging around –85 mV, which were effectively at the Nernst potential for K^+^ ions with this combination of internal and bath solutions. These resting membrane potentials were the same as AOB astroglia (data not shown), and at the extreme end of expectations for neurons in the brain. The goal of this study was not necessarily to interrogate the specific physiologic/biophysical mechanisms for this extreme phenotype, but did suggest that EGCs, at rest, are ∼40 mV hyperpolarized to spike threshold, and may require extensive activation by MCs to fire action potentials. Intriguingly, another feature that distinguished AOB EGCs from other cells was their high rates and amplitudes of spontaneous glutamatergic EPSCs (Figs. 4, 5, 7). This observation, coupled with the dense, spinous dendritic morphology of EGCs, indicates broad and dense innervation by MCs. AOB EGCs, unlike IGCs, possessed small I_H_ sag potentials, and readily sustained 20- to 40-Hz trains of action potentials on moderate somatic depolarization (Figs. 4, 5). This suggests that these cells are capable of distributing broad lateral inhibition among spatially dispersed MCs, but further studies are necessary to investigate this hypothesis.

All three transgenic lines explored in this study labeled EGCs, which are dwarfed in total numbers by IGCs (Fig. 1). EGCs labeled in *Cort-cre* transgenic mice were indistinguishable from *Gad2-cre*-labeled and *Calb2-cre*-labeled EGCs (Fig. 5), but were much less dense than those populations (Fig. 1). This suggests that EGCs labeled in *Cort-cre* transgenic mice represent a subset of the total EGC population. Cortistatin, a somatostatin-like neuropeptide hormone (de Lecea et al., 1996) has never been studied in olfaction; these studies indicate that its role in the AOB may be to modulate MC activity, but further studies are necessary to evaluate this hypothesis.

### AOB JGCs include physiologically distinct subsets associated with marker gene expression

In the MOB, extensive studies into the role of JGCs have identified several JGC subtypes, including PGs and SAs, which play important roles in inter-glomerular and intra-glomerular processes (McGann et al., 2005; Tan et al., 2010; Fukunaga et al., 2014; Livneh et al., 2014). PGs, in general, are thought to support intraglomerular processes, like presynaptic silencing of sensory input, and short-range interglomerular inhibition (for review, see Burton, 2017). SAs, on the other hand, participate extensively in long-range interglomerular inhibition through their activation and inactivation of other cells, including MCs and external tufted cells. Thus, there is abundant evidence for functional diversification among JGCs in the MOB. In the AOB, however, there is very little available information about JGC function (Goldmakher and Moss, 2000; Hu et al., 2016).

The results of our survey of JGCs revealed the presence of two JGC subpopulations with macroscopically similar in their intrinsic physiologic features to PG subsets in the MOB (Puopolo and Belluzzi, 1998; Murphy et al., 2005; Sethupathy et al., 2013; Najac et al., 2015; Burton et al., 2017; Figs. 6, 7). We found that *Gad2*-tdTomato positive cells were nearly all capable of firing sustained action potential trains on moderate depolarization (Fig. 6*B*,*C*). These cells possessed low capacitance and high input resistance, and superficially resemble a faster-spiking populations of MOB PGCs (Puopolo and Belluzzi, 1998; Najac et al., 2015). *Calb2*-tdTomato JGCs demonstrated spontaneous and stimulated plateau potentials, similar to a major class of MOB PGCs (Murphy et al., 2005; Masurkar and Chen, 2011; Fogli Iseppe et al., 2016). We did not explicitly seek out cells with morphologic features matching SAs, but we cannot rule out the possibility that a subpopulation of the regular-spiking *Gad2*-tdTomato JGCs were AOB SAs. In all, these data confirm the presence of physiologically distinct classes of JGCs in the AOB GL. The AOB GL is macroscopically much different in its organization compared to the MOB. The AOB GL has numerous, small glomeruli per vomeronasal receptor input and lacks a clear PG boundary surrounding individual glomeruli (Pinching and Powell, 1971; Belluscio et al., 1999; Rodriguez et al., 1999). As such, the specific computations provided by AOB JGCs are likely to be different from their MOB counterparts. That said, our results indicate that the AOB utilizes JGC subpopulations with similar intrinsic physiologic features to their MOB counterparts, a finding which will support future inquiries into AOB JGC function.

### Implications of current work for models of AOB circuit function

We designed this set of experiments with the simple goal of producing a better quantitative foundation for studying the many populations of interneurons performing inhibition (and perhaps excitation) in the AOB. The results identify functional subdivisions within morphologic types and highlight the need for improved tools for selective labeling of AOB interneurons. By subjecting each of the 150 cells included in these analyses to a simple panel of intrinsic physiologic stimuli, we were able to use multidimensional analysis methods to identify patterns of features that were shared by groups of neurons. These stimulus panels and the physiologic features extracted from the data are easy to implement (taking ∼2 min of time after patching a cell), and can be included in any existing study in which internal patch solutions allow the observation of natural spiking patterns in current clamp (e.g., potassium gluconate-based solutions, etc.). Future studies of AOB interneurons *in vitro* or *in vivo* including these simple intrinsic “fingerprinting” protocols will be able to compare the physiologic profiles observed in each cell to those observed in this study. This approach supports the identification of novel physiologic types or specific changes to established functionally defined types that may occur in response to chemosensory experience (Cansler et al., 2017) and/or neuromodulation (Smith et al., 2009; Smith and Araneda, 2010; Leszkowicz et al., 2012). Taken as a whole, these results provide a major improvement in the available physiologic data for AOB interneurons, and improve the foundation for studies of AOB circuit function.

## Synthesis

Reviewing Editor: Douglas Bayliss, University of Virginia School of Medicine Decisions are customarily a result of the Reviewing Editor and the peer reviewers coming together and discussing their recommendations until a consensus is reached. When revisions are invited, a fact-based synthesis statement explaining their decision and outlining what is needed to prepare a revision will be listed below. The following reviewer(s) agreed to reveal their identity: Marc Spehr, Ian G. Davison.

Synthesis

This manuscript was seen by two reviewers, both of whom found substantial merit in the quality of the data and presentation, and the contribution this work will make to understanding diverse interneuron populations within the accessory olfactory bulb.

The referees provide some relatively minor suggestions for improvement (see below for their full comments). In this respect, please provide additional methodological details for the multidimensional clustering analysis, as well as some discussion of the criteria used to merge and/or subdivide clusters; also, please comment explicitly on how robust the final groupings are in defining functionally distinct interneuron subsets. If relevant data are available, both reviewers also expressed enthusiasm for additional characterization of the morphology of Cort-positive neurons, and the anterior-posterior distribution of Gad2 and Calb2 cells.

Reviewer #1

Advances the Field

The manuscript entitled “Interneuron functional diversity in the mouse accessory olfactory bulb” makes several important contributions to our understanding of the neurobiology of the accessory olfactory system. Most importantly, this study reveals that AOB internal granule cells should not be considered to be a monolithic population. Rather this relatively poorly classified, yet vast interneuron population appears highly heterogeneous.

Using electrophysiological (whole-cell patch-clamp) recordings from genetically labeled interneuron types - i.e., Gad2 (GAD65), Calb2 (calretinin), and Cort (cortistatin)-labeled neurons - in acute AOB slices, the authors of the present study convincingly show that specific intrinsic properties of AOB interneurons do not correspond to any genetically labeled cell type, suggesting that transcriptional heterogeneity among EGCs and IGCs is not correlated with expression of any of these particular marker genes. Importantly, neurons labeled by crossing the Cort-cre transgenic line with a tdTomato reporter strain, are almost completely selective for EGCs in the AOB. This finding identifies this genetic strategy as suitable for selective monitoring or manipulation of this distinct subtype of AOB

interneurons.

The findings presented by the authors are of interest to a wide range of individuals working in the field of chemosensory neuroscience. The data are solid and the authors provide compelling evidence for intrinsic heterogeneity among AOB internal as well as external granule cells (IGCs and EGCs, respectively) and juxtaglomerular cells (JGCs). It is clear that the paper identifies specific physiological features of AOB interneurons types that will assist in future studies of AOB function.

Comments to the Authors

The manuscript entitled “Interneuron functional diversity in the mouse accessory olfactory bulb” makes several important contributions to our understanding of the neurobiology of the accessory olfactory system. Most importantly, this study reveals that AOB internal granule cells should not be considered to be a monolithic population. Rather this relatively poorly classified, yet vast interneuron population appears highly heterogeneous.

Using electrophysiological (whole-cell patch-clamp) recordings from genetically labeled interneuron types - i.e., Gad2 (GAD65), Calb2 (calretinin), and Cort (cortistatin)-labeled neurons - in acute AOB slices, the authors of the present study convincingly show that specific intrinsic properties of AOB interneurons do not correspond to any genetically labeled cell type, suggesting that transcriptional heterogeneity among EGCs and IGCs is not correlated with expression of any of these particular marker genes. Importantly, neurons labeled by crossing the Cort-cre transgenic line with a tdTomato reporter strain, are almost completely selective for EGCs in the AOB. This finding identifies this genetic strategy as suitable for selective monitoring or manipulation of this distinct subtype of AOB interneurons.

The findings presented by the authors are of interest to a wide range of individuals working in the field of chemosensory neuroscience. The data are solid and the authors provide compelling evidence for intrinsic heterogeneity among AOB internal as well as external granule cells (IGCs and EGCs, respectively) and juxtaglomerular cells (JGCs). It is clear that the paper identifies specific physiological features of AOB interneurons types that will assist in future studies of AOB function.

The paper provides an important foundation for follow-up studies and adds substantial novel information about physiologically different interneuron types (and their heterogeneity) in the accessory olfactory bulb. In its present form, the manuscript is basically suitable for publication in eNeuro as is. Very few minor concerns should be addressed before publication (see below).

Minor Points:

a) Discussion; p.18 & 19: “5). Incredibly, AOB EGCs possessed resting membrane potentials, averaging around -85 mV, which were effectively...” & “Intriguingly, another feature that distinguished AOB EGCs from other cells was their incredibly high rates and amplitudes...”

I would not use “incredibly”.

b) Fig. 1C: The relatively sparse labeling of Cort-positive neurons in the ECL allows for a more comprehensive anatomical / morphological description of this still elusive interneuron type. I am aware that this is some work, but I think the authors are missing an opportunity here - i.e., to add very useful information on this specific cell type. If this analysis is available, I would be thrilled to see it added to the manuscript.

c) Fig. 1C: It appears that at the anterior border between AOB and MOB, there are many labeled neurons in the MOB. Any thoughts on that?

d) Fig. 1A-B & Results (p.9): The authors mention that “The pattern of Gad2-tdTomato+ and Calb2-tdTomato+ IGCs varied along the anterior-posterior axis of the AOB, with Gad2-tdTomato+ IGCs enriched in the anterior AOB and Calb2-tdTomato+ IGCs enriched in the posterior AOB. These anterior-posterior biases were most prominent in the lateral-most regions of the AOB.” This is highly intriguing and the authors should comment on that.

Reviewer #2

Advances the Field

Understanding of a neural circuit requires a description of the cellular properties of its component cell types. Careful, systematic analysis of different interneuron classes has been done for many brain areas but data of this type are thin for AOB. This manuscript provides a rich dataset examining the properties of genetically defined cell types that has been lacking to date.

Statistics

The authors use clustering and dimensionality reduction approaches that I have not previously worked with. The analyses appear carefully done and similar methods have been applied in related published work, so there is no indication of any concern, but I don't have enough familiarity to properly assess this part of their analyses.

Comments to the Authors

This manuscript describes a careful electrophysiological characterization of genetically identified cell classes in the accessory olfactory bulb by combining genetic labeling approaches with in vitro whole-cell recordings. The authors focus on three interneuron types, GAD2, calretinin, and cortistatin. They find that each class has distinct localization patterns, but interestingly, also considerable diversity in electrophysiological properties with each type. This work gives a detailed new perspective on the different cellular components of the AOB circuit.

I have no major concerns with the design and main findings of the study. The dataset includes a large set of recordings from each of the different classes and the data are carefully quantified and analyzed.

The main analysis and conclusions rely on a clustering algorithm that groups cells into functional categories using a feature space of 26 characteristics. While the MDS analysis is described elsewhere it forms a major part of the paper and a some more description in the methods would be helpful. The second half of the paper analyzes the full dataset with this approach which reveals a relatively large number of clusters. Some of these look fairly close in MDS space, and some contain a relatively low number of neurons. The large parameter set is a strength of the work, but it is not easy to interpret how functionally distinct these groups really are in practice. The MDS algorithm defines clusters based on statistical variance - how robust are the clustering results over different thresholds for defining a cluster? It would be useful to include some conceptual discussion of the pros and cons of attempting to subdivide neurons into a large number of finely defined categories, vs lumping together more broadly.

Minor Points

In the initial section of the results the authors mention previous work classifying INs by antibody staining. Additional details about the markers used in the work, and how they compare with the markers used by the authors, would add some helpful context.

The authors mention they first screened multiple lines and that they did not find labeling with PV lines. Were other lines also tested? If so some more details could be useful even if results are negative. Regarding the 3 lines used here, is it possible to roughly estimate what fraction of INs are labeled by the 3 markers, and/or their degree of overlap? How fully do the 3 lines cover the full set of interneurons?

The discussion focuses mainly on the potential roles for these cell types in AOB. Are there data available for corresponding cell types in main olfactory bulb? Some comparison between the two structures might be informative. Likewise for similarities or differences with parallel cell classes in other areas like cortex and hippocampus where similar analyses have been done.
